# Comparison of extracorporeal membrane oxygenation outcome for influenza-associated acute respiratory failure in Japan between 2009 and 2016

**DOI:** 10.1186/s40560-018-0306-8

**Published:** 2018-07-11

**Authors:** Shinichiro Ohshimo, Nobuaki Shime, Satoshi Nakagawa, Osamu Nishida, Shinhiro Takeda, Committee of the Japan ECMO project

**Affiliations:** 1 Department of Emergency and Critical Care Medicine, Graduate School of Biomedical and Health Sciences, Hiroshima University, 1-2-3 Kasumi, Minami-ku, Hiroshima, 734-8551 Japan; 2 Department of Critical Care and Anesthesia, National Center for Child Health and Development, Tokyo, Japan; 3 Department of Anaesthesiology and Critical Care Medicine, Fujita Health University School of Medicine, Aichi, Japan; 4 Kawaguchi Cardiovascular and Respiratory Hospital, Saitama, Japan

**Keywords:** Acute respiratory distress syndrome, Mechanical ventilation, Prognosis, Survival, Complication

## Abstract

**Background:**

Since the 2009 pandemic influenza, we have nationally established a committee of the extracorporeal membrane oxygenation (ECMO) project. This project involves adequate respiratory management for severe respiratory failure using ECMO. This study aimed to investigate the correlations between changes in respiratory management using ECMO in Japan and outcomes of patients with influenza-associated acute respiratory failure between 2009 and 2016.

**Methods:**

We investigated the incidence, severity, characteristics, and prognosis of influenza-associated acute respiratory failure in 2016 by web-based surveillance. The correlations between clinical characteristics, ventilator settings, ECMO settings, and prognosis were evaluated.

**Results:**

A total of 14 patients were managed with ECMO in 2016. There were no significant differences in age, sex, and the acute physiology and chronic health evaluation II score between 2009 and 2016. The maximum sequential organ failure assessment score and highest positive end-expiratory pressure were lower in 2016 than in 2009 (*p* = 0.03 and *p* = 0.015, respectively). Baseline and lowest partial pressure of arterial oxygen (PaO_2_)/fraction of inspiratory oxygen (F_I_O_2_) ratios were higher in 2016 than in 2009 (*p* = 0.009 and *p* = 0.002, respectively). The types of consoles, circuits, oxygenators, centrifugal pumps, and cannulas were significantly changed between 2016 and 2009 (*p* = 0.006, *p* = 0.003, *p* = 0.004, *p* < 0.001, respectively). Duration of the use of each circuit was significantly longer in 2016 than in 2009 (8.5 vs. 4.0 days; *p* = 0.0001). Multivariate analysis showed that the use of ECMO in 2016 was an independent predictor of better overall survival in patients with influenza-associated acute respiratory failure (hazard ratio, 7.25; 95% confidence interval, 1.35–33.3; *p* = 0.021).

**Conclusions:**

Respiratory management for influenza-associated acute respiratory failure using ECMO was significantly changed in 2016 compared with 2009 in Japan. The outcome of ECMO use had improved in 2016 compared with the outcome in 2009 in patients with influenza-associated acute respiratory failure.

## Background

Influenza virus can occasionally induce severe respiratory failure, including acute respiratory distress syndrome. The Centers for Disease Control and Prevention reported that more than approximately 20,000 influenza-associated deaths annually occurred in the USA [1]. Extracorporeal membrane oxygenation (ECMO) can be a lifesaving method in patients with potentially reversible acute respiratory failure, including influenza-associated acute respiratory failure [2, 3]. However, Takeda et al. showed that the survival rate of influenza-associated acute respiratory failure managed with ECMO in Japan was inferior compared with that in other countries during the pandemic of H1N1 influenza in 2009 [4–6]. Inadequate use of ECMO equipment (cannula, pump, and oxygenator), insufficient understanding of the ECMO strategy by physicians and other medical staff, and insufficient centralization of ECMO treatment might have affected this poor survival rate in Japan [4].

Since the 2009 pandemic of H1N1 influenza, we have nationally established a committee of an ECMO project, which is expected to guide adequate respiratory management for severe respiratory failure using ECMO. Introduction and simulation education by the ECMO project includes the physiology of ECMO, cannulation techniques, repositioning of the cannula, monitoring skill, daily management, and troubleshooting.

This study aimed to investigate the incidence, severity, characteristics, and prognosis of pandemic of influenza-associated acute respiratory failure that occurred in Japan in 2016. We also aimed to evaluate the correlations between changes in respiratory management using ECMO and outcomes of patients with influenza-associated acute respiratory failure in 2009 and 2016.

## Methods

This study involved adult patients with acute respiratory failure that was associated with H1N1 influenza who were admitted to the institutes of the ECMO project from January to April in 2016. A database was created based on the information collected from the institutes that participated in this study. A total of 87 institutes participate in the ECMO project, and 463 patients with various kind of respiratory failure who underwent ECMO have been registered in the database. Among them, 14 patients in 2009 and 14 patients in 2016 who suffered from influenza-associated acute respiratory failure were analyzed in this study. Data extracted from a previous study [4] were simultaneously analyzed and compared with those in the ECMO 2016 group. Informed consent was obtained from each individual by document or the opt-out procedure. Collected data included baseline characteristics (age, sex, body weight, body temperature, acute physiology and chronic health evaluation [APACHE] II score, and predicted death rate), sequential organ failure assessment (SOFA) score, administered drugs, ventilator settings, ECMO equipment and settings, and outcome. Maximum SOFA score was defined as the highest SOFA score before starting ECMO. Overall survival rate was defined as the survival rate during the follow-up. Inclusion criteria were as follows: (1) patients with influenza-associated acute respiratory failure who were treated in institutes that participated in the ECMO project and (2) age older than 20 years. Categorical differences between the survival and non-survival groups were compared using Fisher’s exact test or the chi-square test. Numerical differences were compared using the Mann–Whitney *U* test. Multivariate analysis was conducted after adjustment for the predicted death rate. All statistical analyses were performed using SPSS software (Abacus Concepts, Berkeley, CA, USA). All values are reported as median (interquartile), and all *p* values less than 0.05 were considered statistically significant. This study was approved by the ethical committee in Hiroshima University with the approval number of E-390-1. Each institute obtained institutional ethics approval and consent to participate.

## Results

### Patients’ characteristics

A total of 14 patients from 16 institutes participating in ECMO project were enrolled as the ECMO 2016 group in this study (Table 1). There were no significant differences in age, sex, weight, body mass index, and APACHE II score between the groups. Maximum SOFA scores in the ECMO 2016 group were significantly lower than those in the ECMO 2009 group. (ECMO 2009 group, 16 [12-19]; ECMO 2016 group, 11 [9-13]; *p* = 0.030). There were no significant differences in the underlying conditions, complications, and the use of rescue and adjunctive therapies (prone positioning, renal replacement therapy, non-invasive positive pressure ventilation) between the groups. The use of peramivir was significantly increased, and the use of oseltamivir was significantly decreased in 2016 compared with 2009. The baseline pressure of arterial oxygen/fraction of inspiratory oxygen ratio (80 vs 55; *p* = 0.009) and the lowest pressure of arterial oxygen/fraction of inspiratory oxygen ratio (70 vs 50; *p* = 0.002) were higher in 2016 compared with 2009. The highest positive end-expiratory pressure was lower in 2016 compared with 2009 (15 vs 24 cmH_2_O; *p* = 0.015). The lowest compliance in 2016 was 31 (9–42) mL/cmH_2_O.

**Table 1:** Baseline characteristics of the patients enrolled

Year	2009	2016	*p* value
*n*	14	14	
Age	54 (43–60)	52 (43–63)	0.70
Sex (male/female)	12/2	12/2	> 0.99
Weight (kg)	70 (64–80)	67 (59–78)	0.50
BMI	NA	23 (22–27)	
Body temperature (°C)			
On admission	38.8 (37.1–39.1)	37.5 (36.7–38.2)	0.11
Maximum	39.4 (38.7–39.8)	38.2 (37.7–39.7)	0.21
APACHE II score	17 (12–25)	20 (5–37)	0.30
Predicted death rate (%)	24.9 (14.6–54.1)	38.0 (34.5–47.8)	0.24
Maximum SOFA score	16 (12–19)	11 (9–13)	0.030
Underlying condition			
Immunosuppression	0 (0)	3 (21)	0.22
Drug abuse	1 (7)	0 (0)	> 0.99
Pregnancy	1 (7)	0 (0)	> 0.99
COPD	0 (0)	2 (14)	0.46
Chronic renal failure	0 (0)	2 (14)	0.46
Vaccination	1 (7)	1 (7)	> 0.99
Influenza antigen/PCR (A/B)	14 / 0	14 / 0	> 0.99
Complications			
Acute renal failure	7 (50)	9 (64)	0.70
Acute hepatic failure	4 (29)	1 (7)	0.32
Culture-confirmed infection	5 (36)	10 (71)	0.13
Shock	4 (29)	5 (36)	> 0.99
Cardiac failure	0 (0)	0 (0)	> 0.99
Respiratory failure	1 (7)	2 (14)	> 0.99
Neurological impairment	0 (0)	0 (0)	> 0.99
Medical treatment			
Peramivir	5 (36)	14 (100)	0.001
Oseltamivir	6 (43)	0 (0)	0.021
Zanamivir	1 (7)	1 (7)	> 0.99
Laninamivir	0 (0)	0 (0)	> 0.99
Antibiotics	13 (93)	13 (93)	> 0.99
gamma-globulin	5 (36)	3 (21)	0.68
Corticosteroid			
High-dose methylprednisolone	9 (64)	6 (43)	0.45
Low dose	7 (50)	6 (43)	> 0.99
Sivelestat	5 (36)	1 (7)	0.17
Vasoactive drugs	13 (93)	11 (79)	0.24
Rescue and adjunctive therapies			
Prone	3 (21)	5 (36)	0.68
Nitric oxide	1 (7)	2 (14)	> 0.99
CRRT	7 (50)	7 (50)	> 0.99
HFOV	0 (0)	0 (0)	> 0.99
APRV	13 (93)	5 (36)	0.006
NPPV	3 (21)	4 (29)	> 0.99
Respiratory impairment before starting ECMO			
PaO_2_/F_I_O_2_ before starting ventilation	55 (46–65)	80 (64–80)	0.009
PaO_2_/F_I_O_2_ at starting ventilation	78 (58–86)	96 (72–150)	0.09
Lowest PaO_2_/F_I_O_2_ during ventilation	50 (41–52)	70 (58–75)	0.002
PEEP at starting ventilation (cmH2O)	10 (10–11)	11 (8–14)	0.48
Highest PEEP during ventilation (cmH_2_O)	24 (17–30)	15 (14–19)	0.015
PIP at starting ventilation (cmH_2_O)	25 (21–29)	21 (18–27)	0.17
Highest PIP during ventilation (cmH_2_O)	30 (30–34)	28 (25–30)	0.10
OI at starting ventilation	NA	16 (3–21)	
Highest OI during ventilation	NA	20 (8–27)	

Data are expressed as median (interquartile) or number (%)

*BMI* body mass index, *APACHE* acute physiology and chronic health evaluation, *SOFA* sequential organ failure assessment, *COPD* chronic obstructive pulmonary disease, *PCR* polymerase chain reaction, *DIC* disseminated intravascular coagulation; *CRRT* continuous renal replacement therapy, *HFOV* high-frequency oscillatory ventilation, *APRV* airway pressure release ventilation, *NPPV* non-invasive positive pressure ventilation, *ECMO*, extracorporeal membrane oxygenation, *PaO*_*2*_*/FIO*_*2*_ pressure of arterial oxygen/fraction of inspiratory oxygen ratio, *PEEP* positive end-expiratory pressure, *PIP* peak inspiratory pressure, *OI* oxygenation index, ICU intensive care unit, *NA* not available

### Changes in ECMO equipment

**Fig. 1:**
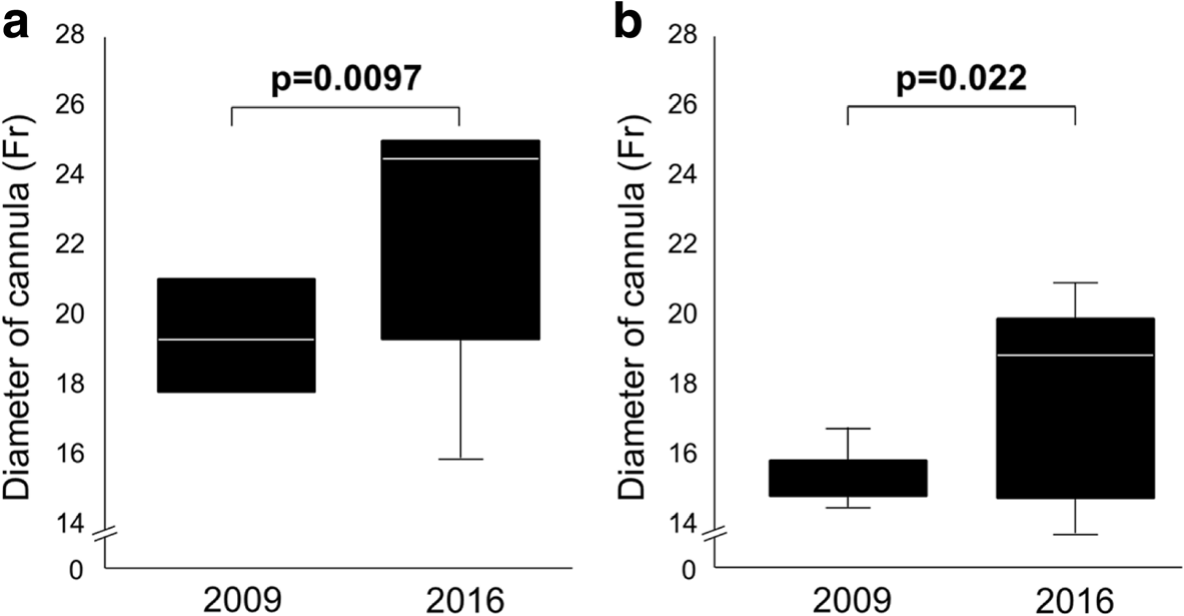
Diameters of the drainage and return cannulas. Box plot graph showing the diameters of **a** the drainage cannula and **b** return cannula used in 2009 and 2016. The diameters of both cannulas were significantly larger in 2016 compared with those in 2009

There were significant changes in the proportions of the console, circuit, oxygenator, and centrifugal pump between 2009 and 2016. The ECMO equipment models used in 2016 widely varied, whereas those in 2009 were almost homogeneous. The diameters of drainage and return cannulas were significantly larger in 2016 compared with 2009 (*p* = 0.0097, *p* = 0.022, respectively; Fig. 1). The durations of each circuit were 4.0 (3.3–4.9) days in 2009, and 8.5 (6.5–14.9) days in 2016, respectively (*p* = 0.0001).

### Approach sites and complications of ECMO

Table 2 shows the approach sites and complications of ECMO. A drainage cannula was inserted in the femoral vein in all of the patients, and a return cannula was inserted into the right jugular vein in 86% of patients in 2009. However, in 2016, the approach sites of drainage and return cannulas were markedly changed. Femoral and right jugular veins became used for either drainage or a return cannula. There were no differences in the incidence of complications, such as oxygenator failure, blood clots, cannula-related problems, pump head complications, massive bleeding, hemolysis, and venous thrombosis. There were also no differences in the incidence of adverse events indirectly associated with the ECMO circuit, such as massive bleeding, hemolysis, disseminated intravascular coagulation, and venous thrombus.

**Table 2:** Approach sites and complications of ECMO

Year	2009	2016	*p* value
Approach site of drainage cannula			0.021
Femoral vein	14 (100)	8 (57)	
Right jugular vein	0 (0)	6 (43)	
Approach site of return cannula			0.0498
Femoral vein	2 (14)	6 (43)	
Right jugular vein	12 (86)	6 (43)	
Femoral artery	0 (0)	2 (14)	
Adverse events directly related to the ECMO circuit			
Oxygenator failure	7 (50)	6 (43)	> 0.99
Blood clots			
Oxygenator	3 (21)	8 (57)	0.12
Other circuit	1 (7)	2 (14)	> 0.99
Cannula-related problems	3 (21)	3 (21)	> 0.99
Pump head complications	1 (7)	1 (7)	> 0.99
Adverse events indirectly related to the ECMO circuit			
Massive bleeding			
Surgical site	4 (29)	3 (21)	> 0.99
Upper digestive tract	4 (29)	2 (14)	0.65
Cannulation site	2 (14)	2 (14)	> 0.99
Pulmonary hemorrhage	1 (7)	0 (0)	> 0.99
Hemolysis	2 (14)	1 (7)	> 0.99
Disseminated intravascular coagulation	10 (71)	5 (36)	0.13
Venous thrombus	2 (14)	4 (21)	0.50

*ECMO* extracorporeal membrane oxygenation

### Outcomes of patients

Outcomes of the patients enrolled are shown in Table 3. Ventilator days before ECMO were shortened from 5.0 days (1.0–7.0 days) to 1.0 day (1.0–2.8 days), but this difference was not significant. Total ventilator days were not significantly different between the ECMO 2016 group and the ECMO 2009 group. Duration of the use of each circuit was significantly longer in 2016 than in 2009 (*p* = 0.0001). There was no difference in the number of patients per institute in 2016 compared with 2009. The length of intensive care unit (ICU) stay was significantly longer in 2016 than in 2009 (*p* = 0.038). The overall survival rate tended to be better in 2016 compared with 2009 (*p* = 0.054).

**Table 3:** Outcomes of the patients enrolled

Year	2009	2016	*p* value
Ventilator days before ECMO (days)	5.0 (1.0–7.0)	1.0 (1.0–2.8)	0.11
Total ventilator days (days)	19 (9–25)	27 (14–38)	0.24
Ventilator-free days (days)	1.5 (0–10.5)	7.5 (4–27)	0.12
Length of ECMO therapy (days)	8.5 (4.5–10.0)	10.0 (8.3–32.5)	0.08
Number of circuits used	2.0 (1.3–3.0)	1.0 (1.0–2.0)	0.14
Duration of each circuit (days)	4.0 (3.3–4.9)	8.5 (6.5–14.9)	0.0001
Number of patients (per institute)	1.0 (1.0–1.0)	1.0 (1.0–1.8)	0.42
Length of ICU stay (days)	17 (9–26)	29 (20–41)	0.038
Length of ICU stay in survived patients (days)	24 (17–26)	24 (20–38)	0.57
Length of hospital stay (days)	25 (12–53)	41 (27–65)	0.14
Length of hospital stay in survived patients (days)	69 (40–77)	42 (23–70)	0.38
Days alive (days)	25 (14–46)	43 (37–73)	0.073
Overall survival rate	5 (36)	11 (79)	0.054
In-hospital survival rate	5 (36)	11 (79)	0.054
60-day survival rate	5 (36)	12 (86)	0.018

Data are expressed as median (interquartile) or number (%)

*ECMO* extracorporeal membrane oxygenation, *ICU* intensive care unit, *NA* not available

### Overall survival

**Fig. 2:**
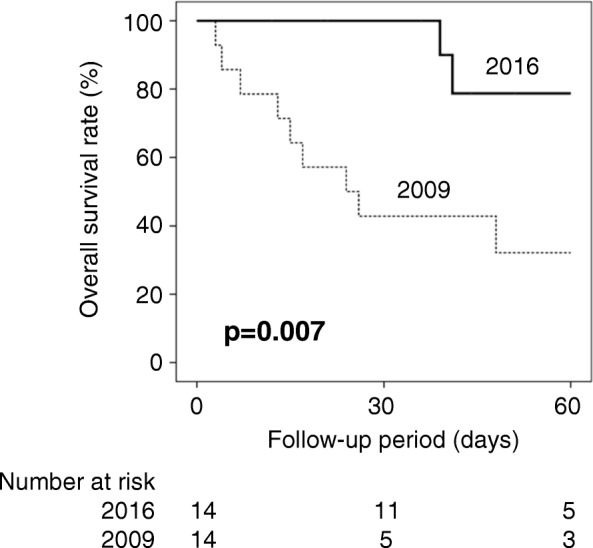
Kaplan–Meier curves for the overall survival rates. Kaplan–Meier curves showing the overall survival rates in each group. The thick solid line indicates the patients in 2016, and the narrow dotted line indicates the patients in 2009. There was a significant difference between the groups (*p* = 0.007)

Kaplan–Meier curves show the overall survival rates in each group (Fig. 2). There was a significant difference between the groups (*p* = 0.007, log-rank test). Univariate analysis demonstrated that the use of ECMO in 2016 (hazard ratio, 6.33; 95% confidence interval [CI], 1.35–33.3; *p* = 0.019) and the maximum SOFA score (hazard ratio, 0.86; 95% CI, 0.76–0.96; *p* = 0.010) were predictive factors of better overall survival. In multivariate analysis, the use of ECMO in 2016 (hazard ratio, 7.25; 95% CI, 1.35–33.3; *p* = 0.021) and the maximum SOFA score (hazard ratio, 0.81; 95% CI, 0.69–0.95; *p* = 0.011) were independent predictive factors of better overall survival (Table 4).

**Table 4:** Univariate and multivariate analyses for better in-hospital and out-of-hospital overall survival

Variable	*β*	HR	(95%CI)	*p* value
Univariate analysis				
Use of ECMO in 2016	1.85	6.33	(1.35–33.3)	0.019
Baseline PaO2/FIO2Baseline PaO2/FIO2	0.05	1.05	(0.98–1.11)	0.15
Lowest PaO2/FIO2Lowest PaO2/FIO2	0.03	1.03	(0.98–1.08)	0.27
Highest PEEP (cmH2O) Highest PEEP (cmH2O)	− 0.07	0.93	(0.85–1.02)	0.12
Maximum SOFA score	− 0.15	0.86	(0.76–0.96)	0.010
Use of peramivir	0.81	2.25	(0.68–7.14)	0.18
Use of oseltamivir	− 0.85	0.43	(0.12–1.47)	0.18
Use of APRV	− 0.36	0.70	(0.18–2.63)	0.60
Size of drainage cannula	− 0.16	0.85	(0.43–1.69)	0.65
Size of return cannula	0.20	1.22	(0.57–2.63)	0.60
Multivariate analysis				
Use of ECMO in 2016	1.98	7.25	(1.35–33.3)	0.021
Maximum SOFA score	− 0.21	0.81	(0.69–0.95)	0.011

*HR* hazard ratio, *CI* confidence interval, *ECMO*, extracorporeal membrane oxygenation, *PaO2/FIO2* partial pressure of arterial oxygen/fraction of inspiratory oxygen ratios, *PEEP* positive end-expiratory pressure, *SOFA* sequential organ failure assessment, *APRV* airway pressure release ventilation

## Discussion

In this study, we showed that the ECMO equipment used for acute respiratory failure in Japan was significantly changed in 2016 compared with 2009. Additionally, the overall survival rate had improved in patients with influenza-associated acute respiratory failure by 2016. Multivariate analysis showed that the use of ECMO in 2016 was an independent predictive factor for a favorable survival.

Takeda et al. showed that the survival rate of patients with 2009 H1N1 influenza-associated respiratory failure managed with ECMO was limited to 36% [4]. They also found that the majority of patients suffered from adverse events associated with the use of ECMO. Previous studies from Europe and Oceania showed the benefit of ECMO for influenza-associated acute respiratory failure [7]. The survival rates in patients with 2009 H1N1 influenza-associated respiratory failure managed with ECMO were 92% in Sweden [6], 76% in the UK [5], 70% in Australia and New Zealand [8], 68% in Italy [9], and 56% in France [10]. Despite the variation in survival rate according to the country, the survival rates in these countries were always better than that in Japan. A recent meta-analysis regarding the benefit of ECMO for influenza-associated acute respiratory failure demonstrated the worst mortality in Japan of 65% compared with the other 12 countries included in this meta-analysis [11]. This finding might be partially explained by the insufficient knowledge and skill in Japan in 2009 regarding ECMO equipment and the relevant physiology.

Based on the insufficient survival rate in 2009, the ECMO project was established in Japan to improve the survival rate of influenza-associated respiratory failure. The educational activity by the ECMO project during these years included the lecture regarding physiology of ECMO, managements of bleeding, coagulation, infection and sedation, selection of pumps, oxygenators and cannulae, and the hands-on simulation for the replacement of oxygenators and total circuits, shift from venovenous to venoarterial ECMO, priming of circuits, and fixing mechanical problems. Activity and education by the committee of the ECMO project in Japan might have changed the strategy of ECMO use and promoted improvement of ECMO management, and consequently, the survival rate in patients with influenza-associated acute respiratory failure had improved in 2016. This was partly reflected by changes in oxygenators and pumps shifting to the long-term durability models, diameters of cannulas shifting to a larger size, and the duration of each circuit use becoming longer. In addition, the number of adverse events was similar in 2009 and 2016 despite the longer duration of each circuit, which might have associated with the improved management of ECMO.

Although the severity of an H1N1 influenza infection that occurred in Mexico and the USA in 2009 was similar to that of seasonal influenza, many patients developed severe respiratory failure that was not typical of conventional seasonal influenza [4]. Our study showed that the severity of influenza-associated acute respiratory failure in Japan in 2016 was similar to that of in 2009 according to the APACHE II score and predicted death rate. Despite the similar severity, we could have significantly improved the survival rate. Recent advances in technology (e.g., biocompatible artificial membranes, heparin-coated circuits, and smaller devices), and network organization with referral ECMO centers have contributed to the dramatic increase in the use of ECMO [12, 13]. However, despite these technological improvements, ECMO is still associated with many complications including bleeding, thrombosis, and nosocomial infection [14–16]. Additionally, in-hospital mortality still remains high (35%–45%), and long-term impairment in physical/psychological function is also significant [16, 17]. Considerable investment for high costs of resources, staffing, and training might be important for improving survival rate and reducing complications. Therefore, improvement in overall survival without any increase in complications observed in this study was impressive. Although this improvement in overall survival might have been associated with an application of ECMO in milder cases, multivariate analysis after adjustment for the SOFA score discounted this possibility.

Baseline and lowest PaO_2_/F_I_O_2_ ratios were higher in 2016 than in 2009, suggesting better oxygenation and lower severity status of the enrolled patients in 2016. In addition, ECMO-associated equipment has been improved in 2016. However, the length of ICU stay was significantly longer in 2016, which was likely to be associated with the improved ability of longer management of ECMO with smaller number of complications.

Bleeding is a serious complication that is associated with ECMO and occurs in approximately 20% of patients [18, 19]. The main mechanisms of bleeding include excessive anticoagulation, thrombocytopenia, and consumption of coagulation factors. Use of ECMO circuits time-dependently activates plasma metalloproteinase-2, a pathway of platelet aggregation, with a subsequent increase in plasma soluble P-selectin concentrations [20]. The resultant platelet dysfunction persists after repeated transfusions of platelets to maintain sufficient platelet counts. Acquired von Willebrand syndrome could also be a complication associated with ECMO. This is characterized by loss of the high molecular weight of von Willebrand factor as a result of shear stress, which impairs binding of von Willebrand factor to platelets [21]. Therefore, adequate selection of ECMO equipment is essential to minimize the shear stress. A shift to the long-term durability models of oxygenators and pumps and to the larger size of diameter of cannulas could have contributed to the reduction in platelet dysfunction, resulting in the longer durability of each circuit in our study.

Previous studies have suggested that centralization of ECMO use to expert referral centers may contribute to improved survival [22, 23]. Propensity score matching analysis suggested that transfer to an ECMO center was associated with a 50% reduction in mortality [5]. Bryner et al. suggested that ECMO centers experiencing more than 30 cases/year were consistently associated with better survival [24]. However, concluding that use of ECMO itself improves survival is difficult because ECMO centers are usually centers of excellence, which can provide better overall intensive care [25]. No definite conclusion can be made because of the lack of randomized, controlled trials. However, adequate use of ECMO after the onset of acute respiratory failure can result in improved survival. Although centralization of ECMO use in Japan has been partially promoted, the number of patients per institute has not yet significantly increased.

Despite the similar severity according to the APACHE II score and predicted death rate, baseline PaO_2_/F_I_O_2_ ratios before and during ventilation were significantly different in both groups. Higher PaO_2_/F_I_O_2_ ratio in 2016 may have associated with less severity in lung injury or early initiation of ECMO. The indication criteria of ECMO are generally considered as patients with mortality risk of 50 to 80%, according to the Extracorporeal Life Support Organization Guidelines [26]. However, the use of ECMO can be associated with various severities of complications, and permissive hypoxemia is an emerging concept in which a lower level of arterial oxygenation can be accepted to avoid the harmful effects of high concentration of inspired oxygen and invasive mechanical ventilation [27]. Therefore, the indication criteria of ECMO might be reconsidered in the future studies.

Our study includes several potential limitations. First, this study was based on surveillance, which did not cover all patients who received ECMO. Second, several clinical items were not completely included because of the retrospective design. Further prospective studies are necessary for confirming the data observed in this study.

## Conclusions

In conclusion, the surveillance in Japan showed that the overall survival rate was significantly improved in patients with influenza-associated acute respiratory failure, who were managed with ECMO in 2016 compared with those in 2009. The procedures of respiratory management using ECMO have significantly changed in 2016. Adequate use of ECMO equipment and promoting better understanding of ECMO physiology in medical staff might have been associated with the improved survival rate in patients with influenza-associated acute respiratory failure in Japan.

## Abbreviations

APACHE: Acute physiology and chronic health evaluation
CI: Confidence interval
ECMO: Extracorporeal membrane oxygenation
F_I_O_2_: Fraction of inspiratory oxygen
PaO_2_: Partial pressure of arterial oxygen
SOFA: Sequential organ failure assessment

## References

[1] Centers for Disease Control and Prevention (CDC) (2010). Estimates of deaths associated with seasonal influenza --- United States, 1976-2007. MMWR Morb Mortal Wkly Rep.

[2] Fan E, Pham T (2014). Extracorporeal membrane oxygenation for severe acute respiratory failure: yes we can! (but should we?). Am J Respir Crit Care Med.

[3] Cooper DJ, Hodgson CL (2013). Extracorporeal membrane oxygenation rescue for H1N1 acute respiratory distress syndrome: equipoise regained. Am J Respir Crit Care Med.

[4] Takeda S, Kotani T, Nakagawa S, Ichiba S, Aokage T, Ochiai R, Taenaka N, Kawamae K, Nishimura M, Ujike Y, Tajimi K (2012). Extracorporeal membrane oxygenation for 2009 influenza a (H1N1) severe respiratory failure in Japan. J Anesth.

[5] Noah MA, Peek GJ, Finney SJ, Griffiths MJ, Harrison DA, Grieve R, Sadique MZ, Sekhon JS, McAuley DF, Firmin RK, Harvey C, Cordingley JJ, Price S, Vuylsteke A, Jenkins DP, Noble DW, Bloomfield R, Walsh TS, Perkins GD, Menon D, Taylor BL, Rowan KM (2011). Referral to an extracorporeal membrane oxygenation center and mortality among patients with severe 2009 influenza a (H1N1). JAMA.

[6] Holzgraefe B, Broome M, Kalzen H, Konrad D, Palmer K, Frenckner B (2010). Extracorporeal membrane oxygenation for pandemic H1N1 2009 respiratory failure. Minerva Anestesiol.

[7] Zangrillo A, Biondi-Zoccai G, Landoni G, Frati G, Patroniti N, Pesenti A, Pappalardo F (2013). Extracorporeal membrane oxygenation (ECMO) in patients with H1N1 influenza infection: a systematic review and meta-analysis including 8 studies and 266 patients receiving ECMO. Crit Care.

[8] Davies A, Jones D, Bailey M, Beca J, Bellomo R, Blackwell N, Forrest P, Gattas D, Granger E, Herkes R, Jackson A, McGuinness S, Nair P, Pellegrino V, Pettila V, Plunkett B, Pye R, Torzillo P, Webb S, Wilson M, Ziegenfuss M (2009). Extracorporeal membrane oxygenation for 2009 influenza a (H1N1) acute respiratory distress syndrome. JAMA.

[9] Patroniti N, Zangrillo A, Pappalardo F, Peris A, Cianchi G, Braschi A, Iotti GA, Arcadipane A, Panarello G, Ranieri VM, Terragni P, Antonelli M, Gattinoni L, Oleari F, Pesenti A (2011). The Italian ECMO network experience during the 2009 influenza a (H1N1) pandemic: preparation for severe respiratory emergency outbreaks. Intensive Care Med.

[10] Roch A, Lepaul-Ercole R, Grisoli D, Bessereau J, Brissy O, Castanier M, Dizier S, Forel JM, Guervilly C, Gariboldi V, Collart F, Michelet P, Perrin G, Charrel R, Papazian L (2010). Extracorporeal membrane oxygenation for severe influenza a (H1N1) acute respiratory distress syndrome: a prospective observational comparative study. Intensive Care Med.

[11] Sukhal S, Sethi J, Ganesh M, Villablanca PA, Malhotra AK, Ramakrishna H (2017). Extracorporeal membrane oxygenation in severe influenza infection with respiratory failure: a systematic review and meta-analysis. Ann Card Anaesth.

[12] MacLaren G, Combes A, Bartlett RH (2012). Contemporary extracorporeal membrane oxygenation for adult respiratory failure: life support in the new era. Intensive Care Med.

[13] Blum JM, Lynch WR, Coopersmith CM (2015). Clinical and billing review of extracorporeal membrane oxygenation. Chest.

[14] Schmidt M, Zogheib E, Roze H, Repesse X, Lebreton G, Luyt CE, Trouillet JL, Brechot N, Nieszkowska A, Dupont H, Ouattara A, Leprince P, Chastre J, Combes A (2013). The PRESERVE mortality risk score and analysis of long-term outcomes after extracorporeal membrane oxygenation for severe acute respiratory distress syndrome. Intensive Care Med.

[15] Cooper E, Burns J, Retter A, Salt G, Camporota L, Meadows CI, Langrish CC, Wyncoll D, Glover G, Ioannou N, Daly K, Barrett NA (2015). Prevalence of venous thrombosis following Venovenous extracorporeal membrane oxygenation in patients with severe respiratory failure. Crit Care Med.

[16] Zangrillo A, Landoni G, Biondi-Zoccai G, Greco M, Greco T, Frati G, Patroniti N, Antonelli M, Pesenti A (2013). Pappalardo F. A meta-analysis of complications and mortality of extracorporeal membrane oxygenation. Crit Care Resusc.

[17] Rozencwajg S, Pilcher D, Combes A, Schmidt M (2016). Outcomes and survival prediction models for severe adult acute respiratory distress syndrome treated with extracorporeal membrane oxygenation. Crit Care.

[18] Munshi L, Telesnicki T, Walkey A, Fan E (2014). Extracorporeal life support for acute respiratory failure. A systematic review and metaanalysis. Ann Am Thorac Soc.

[19] Ried M, Bein T, Philipp A, Muller T, Graf B, Schmid C, Zonies D, Diez C, Hofmann HS (2013). Extracorporeal lung support in trauma patients with severe chest injury and acute lung failure: a 10-year institutional experience. Crit Care.

[20] Cheung PY, Sawicki G, Salas E, Etches PC, Schulz R, Radomski MW (2000). The mechanisms of platelet dysfunction during extracorporeal membrane oxygenation in critically ill neonates. Crit Care Med.

[21] Heilmann C, Geisen U, Beyersdorf F, Nakamura L, Benk C, Trummer G, Berchtold-Herz M, Schlensak C, Zieger B (2012). Acquired von Willebrand syndrome in patients with extracorporeal life support (ECLS). Intensive Care Med.

[22] Barbaro RP, Odetola FO, Kidwell KM, Paden ML, Bartlett RH, Davis MM, Annich GM (2015). Association of hospital-level volume of extracorporeal membrane oxygenation cases and mortality. Analysis of the extracorporeal life support organization registry. Am J Respir Crit Care Med.

[23] Peek GJ, Mugford M, Tiruvoipati R, Wilson A, Allen E, Thalanany MM, Hibbert CL, Truesdale A, Clemens F, Cooper N, Firmin RK, Elbourne D (2009). Efficacy and economic assessment of conventional ventilatory support versus extracorporeal membrane oxygenation for severe adult respiratory failure (CESAR): a multicentre randomised controlled trial. Lancet.

[24] Bryner B, Cooley E, Copenhaver W, Brierley K, Teman N, Landis D, Rycus P, Hemmila M, Napolitano LM, Haft J, Park PK, Bartlett RH (2014). Two decades’ experience with interfacility transport on extracorporeal membrane oxygenation. Ann Thorac Surg.

[25] Wallace DJ, Milbrandt EB, Boujoukos A (2010). Ave, CESAR, morituri te salutant! (hail, CESAR, those who are about to die salute you!). Crit Care.

[26] Extracorporeal Life Support Organization (ELSO). General Guidelines for all ECLS Cases. https://www.elso.org/Portals/0/ELSO%20Guidelines%20General%20All%20ECLS%20Version%201_4.pdf. Accessed 30 May 2018.

[27] Gilbert-Kawai ET, Mitchell K, Martin D, Carlisle J, Grocott MP (2014). Permissive hypoxaemia versus normoxaemia for mechanically ventilated critically ill patients. Cochrane Database Syst Rev.

